# Role of Galanin system and insulin resistance parameters as predictive tools for diagnosis of Long-COVID patients

**DOI:** 10.1016/j.bbrep.2025.102068

**Published:** 2025-06-03

**Authors:** Wasim Talib Al Masoodi, Sami Waheed Radhi, Habiba Khdair Abdalsada, Hussein Kadhem Al-Hakeim

**Affiliations:** a Department of Chemistry, College of Science, University of Kufa, Najaf, Iraq; b Department of Chemistry, Faculty of Medicine, University of Al-Ameed, Karbala, 56001, Iraq; c College of Pharmacy, Al-Muthanna University, Iraq

**Keywords:** Long-COVID, Galanin, Galanin receptor, Neuronal network, Gal/GalR-1, Insulin resistance

## Abstract

**Background:**

COVID-19 patients may have long-lasting symptoms known as long-COVID (LC) without any underlying medical issues or obvious organ damage. Much research suggested that these issues are attributed to cytokine storm, lung and nerve injury, and glucose homeostasis disruption. Galanin (Gal), a neuropeptide in the peripheral and central nervous systems, has several physiological activities connected to illnesses. The current case-control research hypothesized the role of insulin resistance (IR) and the Gal system in LC pathophysiology.

**Methods:**

This research included 30 healthy controls and 60 LC patients. Insulin, Gal, and GalR1 were determined using the enzyme-linked immunosorbent assay (ELISA). The HOMA2 calculator determined β-cell function (HOMA%B), insulin sensitivity (HOMA%S), and insulin resistance (HOMA2IR) by analyzing fasting serum insulin and glucose levels.

**Results:**

LC patients showed higher Gal, GalR1, and Gal/GalR1 concentrations than controls, suggesting Gal system activation. LC patients likely have an IR state. The correlation study showed a negative link between Gal, GalR1, and SpO2. Gal level was positively correlated with insulin, insulin/glucose, and HOMA2IR and negatively correlated with HOMA%S. With an AUC-ROC of 0.939, artificial neural networks (ANN) predicted a sensitivity of 71.4 % and a specificity of 87.5 %. In LC, IR parameters and Gal system biomarkers were strongly correlated, suggesting they may contribute to disease.

**Conclusion:**

Galanin system and IR parameters are altered in LC patients and can predict LC in suspicious subjects with 91.7 % sensitivity and 100.0 % specificity using the neural network model. The top five predictors were CRP, insulin/glucose, Gal, glucose, and GalR1. CRP had the greatest importance (100.0 %), indicating the importance of inflammation, IR, and Gal system biomarkers in the pathophysiology of LC.

## Highlights

• Long-COVID patients had higher galanin and its receptor (GalR1) than the controls.
• Biomarker analysis by artificial neural network significantly predicts Long COVID.
• C-Reactive Protein had the strongest association with Long COVID severity.
• Insulin resistance state is highly prevalent in Long COVID patients.
• Galanin system and insulin resistance are involved in Long COVID pathophysiology.

## Graphical abstract

**The overall results of the comparisons of biomarkers levels between Long-COVID and controls, major correlations between biomarkers, and artificial neural networks (ANN) analysis**.*CRP: C-reactive protein, Galanin: Gal, GalR1: Galanin Receptor-1, Gal/GalR1: Galanin to Galanin Receptor-1, HOMA2IR: Homeostatic Model Assessment 2 (HOMA2) of Insulin Resistance (IR), HOMA2 %B: HOMA2 Beta-cell Function, HOMA2 %S: HOMA2 Insulin Sensitivity, and I/G: Insulin/Glucose ratio.*

**Figure:**
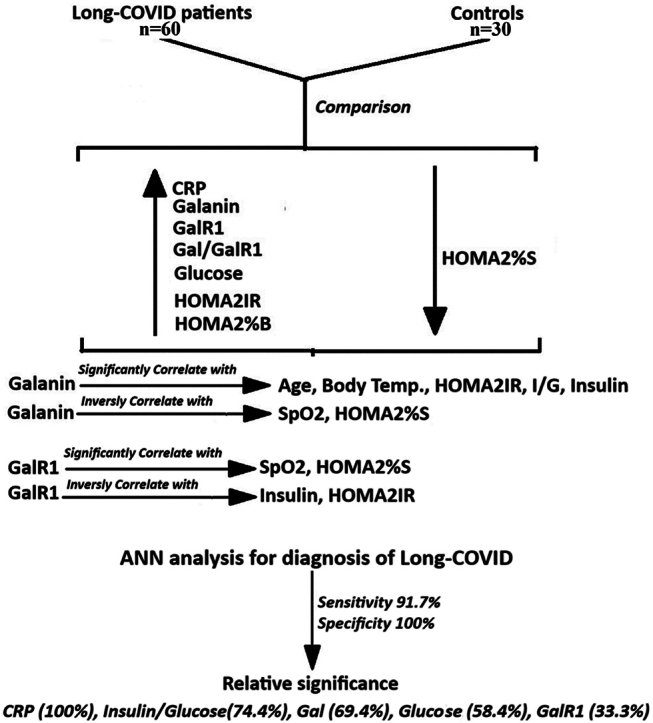

## 1 Introduction

Long COVID (LC) is a post-COVID-19 condition characterized by persistent symptoms lasting at least two months and not explained by alternative diagnoses, typically occurring three months after the onset of COVID-19 [1]. A variety of persistent and varied symptoms are seen with this complex illness [2]. During the acute period of COVID-19 hospitalization, patients were more likely to have symptoms such as fatigue, memory loss, and breathing complications, with the latter two being reported more commonly than the prior two [3]. The hypothesis regarding the appearance of LC symptoms and its pathophysiology is still a subject of debate, though several hypotheses have been proposed to explain different aspects of this complex condition [[4], [5], [6]]. The persistence of SARS-CoV-2, direct organ damage, perturbations in the innate and adaptive immune systems, autoimmunity, latent viral reactivation, endothelial dysfunction, and abnormalities in the microbiome are critical factors for understanding the evolution, complexity, and causes of LC [[4], [5], [6]]. Although initial research suggested the persistence of RNA of SARS-CoV-2 [7], a recent study does not support the persistence of the virus [8]. Another finding showed that LC patients have immune cell activation and increased levels of organ-damage-associated proteins [9]. LC has been shown to be associated with autoimmunity abnormalities, including cross-reactive T-cells, antigen-specific tissue resident memory cells, and autoantibodies [10]. There are also many reports about the immune dysregulation in LC [[11], [12], [13]] expressed as increased activated memory CD4 + T cells, neutrophils, and inflammatory biomarkers [11,14]. These mechanisms are further enforced by the elevated levels of the terminal effectors of CD4 and CD8, monocyte chemoattractant protein-1, and CD71^+^ erythroid cells, and soluble CD14 protein in LC patients [13,14]. Also, persistent complement dysregulation and thromboinflammation are other explanations for the LC [15]. Many studies have investigated the functions of different substances in diagnosing the causes and monitoring the progression of LC. The Galanin (Gal) system markers have not been examined in the context of LC. The neuropeptide Gal, comprising 29–30 amino acids, is present in the peripheral and central neural systems and has diverse physiological roles [16]. The Gal system is linked to a variety of disorders in humans, including pain, depression, epilepsy, neurodegenerative diseases, diabetes, and cancer [17]. Also, it is activated in inflammation [18] and viral infections such as retrovirus [19] and SARS-CoV-2 [20]. These conditions are presented in the LC and provide the rationale to study the Gal system in LC. Hence, understanding the role of the Gal system in LC can assist in formulating diagnostic and thereby strategies for treatment of this complex condition. Research has indicated that individuals with pre-existing medical conditions such as diabetes or obesity are more prone to experiencing persistent symptoms of COVID-19, including fatigue, difficulty breathing, and muscle pain [21]. Furthermore, it has been determined that clinical risk factors and sociodemographic characteristics play a significant role in influencing the severity of COVID-19 [22]. Additionally, a study focused on LC in youth found that the diagnosis of LC syndrome in this population was influenced by clinical and sociodemographic characteristics [23]. These findings highlight the need to consider clinical risk factors and sociodemographic factors for detecting and diagnosing LC.

Gal is essential for maintaining the metabolic balance of glucose [24] Since it correlates with body mass index (BMI), glucose tolerance test (GTT), and fasting blood glucose [24,25]. Gal is correlated with insulin and TG [26], in addition to the findings that Gal increased in subjects with impaired glucose tolerance tests [27]. These relations encouraged us to study Gal and IR in LC patients. Insulin resistance (IR) has been identified as a potential prognostic sign in the assessment of acute COVID-19 [28]. Furthermore, it serves as a significant indicator of both severe acute COVID-19 and protracted COVID-19, making it a crucial risk factor [29]. COVID-19 can lead to the development of IR, a metabolic state that can persist even after the virus has been eliminated [30]. Pre-existing metabolic illnesses, such as obesity, type 2 diabetes, and cardiovascular disease, have been linked to a higher likelihood of developing LC symptoms [31]. Obese patients with COVID-19 experience unfavorable outcomes, primarily due to IR, regardless of their body mass index (BMI) [32]. Furthermore, in patients with diabetes and obesity, the combination of hormonal resistance associated with obesity and the IR caused by COVID-19 exacerbates the severity of illness [33]. Studying the Gal System and IR measurements is essential for LC prediction and diagnosis since it advances our knowledge of the pathophysiology of the illness. With biomarkers, Artificial Neural Network (ANN) can monitor therapy response, assess disease severity, adapt treatment regimens, enable early detection, and improve patient outcomes [34].

Artificial neural networks (ANNs) have demonstrated the potential to enhance epidemic containment tactics by accurately predicting COVID-19 infections [35]. Furthermore, ANN models have exhibited precision in predicting the overall vital status post-hospital discharge [36]. The classification of patients, both with and without severe disease, can be effectively achieved using ANN by applying the accuracy statistic known as the area under the receiver operating characteristic (ROC) [37]. These results show the possibility and potential of ANN study in the design of dependable LC diagnostic instruments. In this work, the relation among LC, IR measures, and the Gal system was studied using a prospective observational methodology. LC patients who met specific inclusion criteria were nominated as participants, and comprehensive data assembly was shown, containing blood samples to measure Gal and GalR1 levels, receptor expression, and IR parameters. To examine the association between these parameters and LC, statistical diagnostic methods such as regression analysis and correlation were applied. The objective of this approach was to study the possible alteration in the Gal system, IR, and inflammation of LC. Also, the potential predictability of this system of LC is well-analyzed.

## 2 Subjects and methods

### 2.1 Participants

The current case-control study enrolled 60 LC patients and 30 control participants. The World Health Organization's criteria for post-COVID cases were used to identify LC patients [38]. They qualify as patients: (a) Confirmed SARS-CoV-2 infection; (b) Post-acute symptoms or symptoms during COVID-19 recovery; (c) experience persistent symptoms lasting for a minimum of two months and continuing three to four months after the start of COVID-19; and (d) at least two symptoms that make daily chores difficult, such as exhaustion, headaches, fever, difficulty speaking, a persistent cough, chest pain, loss of taste or smell, mental impairment, or emotional problems [38]. The study included thirty healthy controls who previously recovered from COVID-19 without the presence of any symptoms of LC. At a hospital in Kerbala city designated as a quarantine, all patients diagnosed with acute COVID-19 were treated. The patients were previously treated for acute COVID-19 infection in the Imam Al-Hussein Medical City, the Imam Al-Hassan Al-Mujtaba Teaching Hospital, and Al-Kafeel Super Specialty Hospital in Kerbala governorate, Iraq. Senior virologists' doctors correctly identified a SARS-CoV-2 infection and acute COVID-19 based on fever, coughing, trouble breathing, and loss of smell and taste, as well as positive results from a reverse transcription real-time polymerase chain reaction and IgM directed against SARS-CoV-2. All patients' rRT-PCR findings were negative after the acute period. The study excluded people with immunity problems, liver disease, neurodegenerative or neuroinflammatory disorders, diabetes mellitus, or other systemic autoimmune diseases. Furthermore, the study excluded women who were pregnant or breastfeeding. All participants gave written consent before participation in the study after being fully informed. The research was approved by the Kerbala Health Directorate-Training and Human Development Center (Document No. 30/2023) and the University of Kufa's institutional ethics committee (1657/2023). The research complied with ethical and privacy regulations in Iraq and abroad, such as the World Medical Association's Helsinki Declaration, the Belmont Report, the International Conference on Harmonization of Good Clinical Practice, and the CIOMS Guidelines. Furthermore, our institutional review board adheres to the International Guidelines for the Safety of Human Research (ICH-GCP). The BMI was determined by dividing the weight in kilograms by the square of the height in meters.

### 2.2 Assays

A five-milliliter of fasting venous blood samples were collected in the early morning and transferred into clean, empty tubes. Any samples that exhibited hemolysis were eliminated from subsequent examination. Once the clotting time was up, the blood samples were spun at 3000 rpm for five minutes. Afterward, the serum was isolated and put into three fresh Eppendorf tubes for storage at −80 °C until analysis. The ELISA kits provided by Nanjing Pars Biochem Co., Ltd. (Nanjing, China) were used to measure the levels of serum Gal, using Gal ELISA kits (Cat No. PRS-00645hu), and GalR1 using ELISA kits (Cat No. PRS-029925hu). The Gal and GALAR1 assays demonstrated an intra-assay coefficient of variation (CV) of 10.0 % (precision within the same assay). CRP latex slide tests from Spinreact® (Barcelona, Spain) measured serum CRP. The concentrations of albumin and glucose in serum were measured using a spectrophotometric method with a commercially available kit provided by Spinreact® (Barcelona, Spain). We rigorously complied with the manufacturer's instructions, meticulously executing each step. Homeostasis Model Assessment 2 (HOMA2) calculator© (Diabetes Trials Unit, University of Oxford; https://www.dtu.ox.ac.uk/homacalculator/download.php) was used to calculate β-cell function (HOMA%B), insulin sensitivity (HOMA%S), and IR (HOMA2-IR) from the fasting serum insulin and glucose levels. Moreover, based on the HOMA calculator software, patients with clear major overt diabetic complications such as heart, liver, and renal problems and fasting insulin >400 pM were excluded. Furthermore, given that metformin may affect insulin sensitivity and IR [39], excluded from our study were people who were taking metformin [40]. This study excluded women who were pregnant or breastfeeding.

### 2.3 Statistical analysis

The distribution of the variables was examined utilizing the Kolmogorov-Smirnov test. The mean standard deviation was employed to represent the outcomes of the variable that followed a normal distribution. The results of the nonparametric variables were expressed as medians and interquartile ranges (25th to the 75th percentile). The analysis of variance (ANOVA) was employed to compare the continuous variables that are normally distributed, while the Chi-square (χ^2^) test was utilized for comparison between nominal variables. Pearson's product-moment correlation coefficients were used to investigate the relationship between the two variables. The Mann-Whitney *U* test was employed to compare the measurement parameters between the control and patient groups. By calculating Spearman's correlation coefficients (rho), we may approximate the level of correlation between the parameters. The associations between categories and biomarkers were assessed using a univariate general linear model (GLM) analysis, which accounted for potential confounding variables such as smoking, sex, age, BMI, and education. Receiver operating characteristics (ROC) analysis was employed to evaluate the diagnostic efficacy of the identified biomarkers. Youdin's statistics, cut-off points, sensitivities, and specificities are calculated variables. SPSS version 26 was used for two-tailed testing with a 0.05 p-value. Excel 2021 from Microsoft Office was used to organize the data.

## 3 Results

### 3.1 Sociodemographic and clinical data

The sociodemographic and clinical attributes of acute COVID-19 patients are divided into two groups: Table 1 displays those who are asymptomatic for LC and those who have symptoms of LC. There were no significant differences in the demographic characteristics of LC patients and healthy controls, including age, sex, BMI, marital status ratio, smoker status, vaccination history (A, PF, S), and treatment history (paracetamol, azithromycin, VitC, VitD, and Zn treatment). LC patients showed significant increases in the duration of disease, maximum body temperature, duration of disease in days, period of cure, and administration of Ceftriaxone, Dexamethasone, Clexane, and Bromhexine. While the patients' group had a significantly lower SpO2 level compared to the control group.

**Table 1:** Socio-demographic characteristics of long-COVID patients and control groups.

Parameters		Controls	Patients	F/χ^2^	df	p
Age Yrs.		33.23 ± 6.11	35.97 ± 9.17	2.176	1	0.144
BMI Kg/m^2^		27.16 ± 2.83	27.45 ± 4.36	0.107	1	0.744
Sex M/F		10/20	24/36	2.829	1	0.093
Single/Married		5/25	5/55	1.406	1	0.236
Smoking No/Yes		15/15	42/18	3.445	1	0.063
Vaccination A1,PF2,S3		9/7/14	26/6/28	4.077	3	0.253
Duration of disease days		7.00(4.75–14.00)	14.00(10.00–15.00)	–	–	<0.001
Period of Cure Months		7.50(5.00–14.00)	30.00(14.00–30.00)	–	–	<0.001
Highest temp. °C		38.00(38.00–40.00)	39.00(38.00–40.00)	–	–	0.005
SpO2 %		96.00(92.25–98.00)	85.00(70.00–90.00)	–	–	<0.001
Paracetamol No/Yes		3/27	5/55	0.069	1	0.793
Ceftriaxone No/Yes		1/29	32/28	16.604	1	<0.001
Dexamethasone		27/3	36/24	8.079	1	0.004
Azithromycin No/Yes		7/23	13/47	0.032	1	0.858
Vitamin C	No/Yes	10/20	13/47	1.431	1	0.232
Vitamin D	No/Yes	11/19	16/44	0.952	1	0.329
Zn treatment No/Yes		12/18	16/44	1.659	1	0.198
Clexane	No/Yes	3/27	41/19	5.084	1	0.024
Bromhexin	No/Yes	3/27	38/22	6.847	1	0.009

Results expressed as mean ± standard deviation for normally distributed data and median (25 %–75 % interquartile) for abnormally distributed variables. Binomial data were expressed as ratios and analyzed by the Chi-squared test. A, PF, S (brands of COVID-19 vaccine): AstraZeneca, PF, Pfizer, and Sinopharm, BMI: body mass index, SpO2: Oxygen saturation percentage, F/χ2: F-statistics value for continuous variables or Chi-square statistic value for categorial variables, df: degree of freedom between groups/within groups, p: probability value.

### 3.2 Comparison of the measured biochemicals

**Fig. 1:**
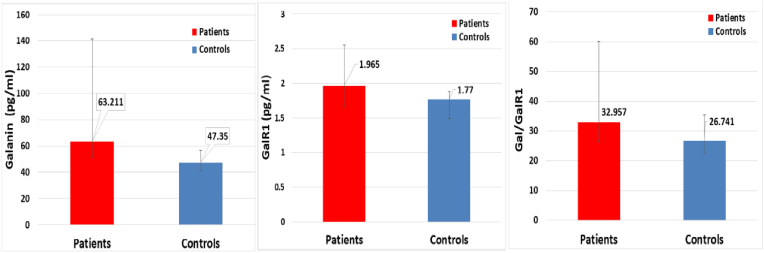
Serum galanin (Left), galanine receptor 1 (GALR1) (Middle), and galanin/GALR1 (Right) in long-COVID patients and control groups.

The comparison of Gal and its receptor and IR parameters between LC patients and controls is shown in Table 2. There is a significant increase in serum Gal, GalR1, and the Gal/GalR1 ratio in comparison to the control group as seen in Fig. 1. Furthermore, the LC patients showed statistically significant increases in HOMA2IR, HOMA%B, and Glucose when compared to the control group. There is a notable reduction in HOMA%S when compared to the control group. Nevertheless, there was no discernible variation in the Ins/Glu between the control group and the LC patients (p = 0.535).

**Table 2:** Comparison of the galanin and its receptor and insulin resistance parameters between Long-COVID patients and controls.

Parameters	Controls	Patients	p
Galanin pg/ml	47.35(41.367–57.08)	63.21(51.58–141.62)	<0.001
GalR1 (pg/ml)	1.77(1.487–1.88)	1.97(1.66–2.55)	0.007
Gal/GalR1	26.74(22.65–35.40)	32.96(26.63–60.06)	0.019
Glucose mM	5.14 ± 0.32	6.01 ± 1.18	<0.001
Insulin pM	72.70 ± 24.70	106.84 ± 34.70	<0.001
HOMA2IR	1.45(1.01–1.75)	2.08(1.65–2.56)	<0.001
HOMA2 %B	109.05 ± 23.96	114.02 ± 40.24	0.002
HOMA2 %S	69.05(57.03–99.78)	48.00(39.05–60.70)	<0.001
Ins/Glu	14.05 ± 4.51	18.17 ± 6.02	0.535

Galanin, Galanin Receptor-1, Galanine to Galanin Receptor-1 ratio, Insulin resistance(IR), the homeostasis model assessment of insulin resistance (HOMA-IR) characteristics of LC patients and HC control expressed as median (25 %–75 % interquartile) and compared by Mann-Whitney U test.

### 3.3 Correlation between gal and its receptor with other parameters

The correlation study identified multiple significant associations between Gal, GalR1, and their ratio with other parameters and serum biomarkers in LC patients are presented in Table 3. The findings demonstrated an inverse relationship between Gal levels with SpO2 (ρ = −0.295, p < 0.01), and HOMA%S (ρ = −0.394, p < 0.01). While there is a significant correlation between Gal with the highest temperature during the acute phase of COVID-19 infection (ρ = 0.201, p < 0.05), age (ρ = 0.353, p < 0.01), I/G (ρ = 0.328, p < 0.01), insulin (ρ = 0.386, p < 0.01), and HOMA2IR (ρ = 0.394, p < 0.01).

**Table 3:** Correlations between biomarkers and socio-demographic characteristics in patients.

	Galanin	GalR1	Gal/GalR1
Sex	0.013	−0.021	0.075
Age	**0.353**^b^	0.124	**0.218**^a^
Weight	0.126	0.022	0.145
Height	−0.015	−0.012	0.031
BMI	0.153	−0.002	0.143
Mar. State	0.106	0.053	0.093
Education years	−0.042	−0.144	0.086
Vaccination	0.051	−0.125	0.132
Smoking	−0.009	0.169	−0.099
Residency	−0.051	−0.139	−0.003
Employment	−0.066	−0.072	−0.018
Duration of Dis.	−0.006	−0.020	0.064
Period of Cure	0.048	0.130	−0.022
Highest temp.	0.201	0.134	0.048
SpO2	**−0.295**^b^	**−0.232**^a^	−0.106
I/G	**0.328**^b^	0.201	0.096
Glucose	0.159	0.203	0.037
Insulin	**0.386**^b^	**0.259**^a^	0.135
HOMA%B	0.164	0.121	−0.001
HOMA%S	**−0.394**^b^	**−0.269**^a^	−0.143
HOMA2IR	**0.394**^b^	**0.269**^a^	0.143
CRP	0.158	0.121	0.059

a Correlation is significant at the 0.05 level (2-tailed).

b Correlation is significant at the 0.01 level (2-tailed).

GalR1 has a significant inverse relationship with SpO2 (ρ = −0.232, p < 0.05), and HOMA%S (ρ = −0.269, p < 0.05). While a significant correlation was found between GalR1 with insulin (ρ = 0.259, p < 0.05), and HOMA2IR (ρ = 0.269, p < 0.05).

There was a significant association between Gal/GalR1 levels and Age (ρ = 0.218, p < 0.05). Other biomarkers have no significant correlations with the Gal, GalR1, and its ratio.

### 3.4 Multivariate generalized linear model (GLM) analysis

The effects of the diagnosis were evaluated using a multivariate General Linear Model (GLM) analysis that accounted for several confounding variables, including sex, age, BMI, highest temperature, SpO2, and smoking, as shown in Table 4. The findings indicate that the diagnosis of LC in a patient has a significant effect on the levels of the measured biomarkers (F = 4.146, p = 0.001) with the highest size effect (partial η^2^ = 0.388). The cofounders (smoking, sex, age, BMI, highest temperature, and SpO2) have no significant impact (p > 0.05) on the biomarker levels. Based on these data, it appears that having LC or being a patient is the main factor impacting the serum levels of the biomarkers. While other confounding variables included in the investigation do not significantly affect changes in the biomarker levels, the presence of LC does.

**Table 4:** Results of the multivariate generalized linear model (GLM) analysis and the between-subjects effects tests of the Long COVID on the biomarkers.

Test	Dependent Variable	Effect	F	p	Partial η^2^
**Multivariate Tests**	**All measured parameters**	Diagnosis	4.146	< **0.001**	**0.388**
		BMI	1.470	0.162	0.183
		Age	1.397	0.193	0.176
		Highest temp.C	1.089	0.382	0.143
		Sex	0.703	0.731	0.097
		Smoking	0.643	0.786	0.089
		SpO2	0.555	0.858	0.078
**Tests of Between-Subjects Effects**	**Diagnosis**	HOMA2IR	11.296	**0.001**	**0.121**
		HOMA%S	10.773	**0.002**	**0.116**
		Insulin	9.825	**0.002**	**0.107**
		Glucose	8.662	**0.004**	**0.096**
		CRP	6.659	**0.012**	**0.075**
		GalR1	6.078	**0.016**	**0.069**
		Galanin	5.256	**0.024**	**0.060**
		I/G	3.592	0.062	0.042
		GalR1/Gal	2.527	0.116	0.030
		HOMA%B	0.035	0.852	0.001

F:F-statistics value for continuous variables, df: degree of freedom, p: probability value, Partial η^2^: Effect size, CRP: C-reactive protein, GalR1: Galanin Receptor-1, Gal/GalR1: Galanin to Galanin Receptor-1, HOMA2IR: Homeostatic Model Assessment (HOMA) of insulin resistance, HOMA%B: HOMA of Beta-cell Function, HOMA%S: HOMA of insulin sensitivity.

The between-subject effects tests demonstrated that the inclusion of LC patients had a substantial impact on multiple parameters. Out of all the factors examined, the HOMA2 IR level was the most strongly impacted, with the LC diagnosis explaining 12.1 % of the variation in HOMA2IR (partial η^2^ = 0.121). Other biomarkers that were significantly affected were HOMA%S (partial η^2^ = 0.116), insulin (partial η^2^ = 0.107), glucose (partial η^2^ = 0.096), CRP (partial η^2^ = 0.075), GalR1 (partial η^2^ = 0.069), and Gal (partial η^2^ = 0.060).

### 3.5 Effect of drugs taken on LC patients

The outcomes of the multivariate GLM analysis, which evaluates the impact of drugs taken on the measured parameters, are displayed in Table 5. The analysis assessed the influence of drugs on levels of biomarkers while accounting for other factors. The findings suggest that the administration of medications did not have a statistically significant impact on the levels of biomarkers (p > 0.05).

**Table 5:** Results of the multivariate generalized linear model (GLM) analysis for estimating the effect of drugs taken of the measured biomarkers in the Long COVID patients.

Test	Dependent Variable	Effect	F	p	Partial η^2^
**Multivariate Tests**	All measured parameters	Cefteriaxone	2.451	0.054	0.126
		Dexamethasone	1.686	0.059	0.125
		Bromhexin	1.641	0.069	0.123
		Clexane	1.303	0.210	0.107
		Azthromycin	1.274	0.229	0.106
		Paracetamol	0.808	0.707	0.077
		Vitamin D	0.785	0.734	0.075
		Zinc	0.660	0.862	0.066
		Vitamin C	0.597	0.912	0.061

F: F-statistics value for continuous variables, df: degree of freedom, p: probability value, Partial η^2^: Effect size.

### 3.6 Artificial neuronal network (ANN) analysis

**Fig. 2:**
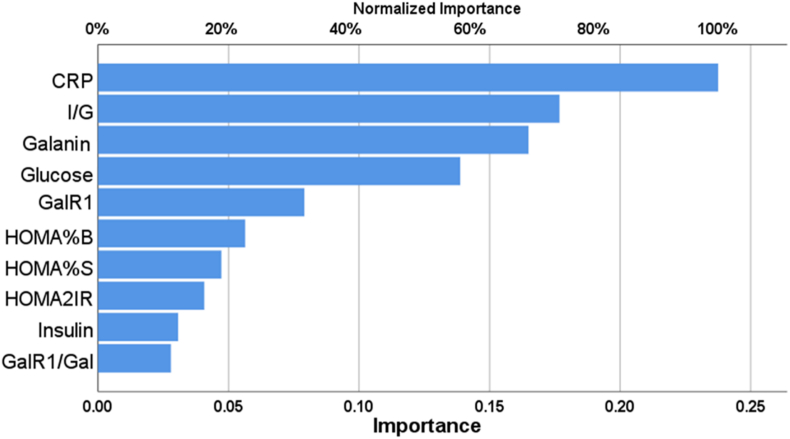
The importance of biomarkers in distinguishing between long-COVID and the control group using artificial neural network analysis. *CRP: C-reactive protein, I/G: insulin-to-glucose ratio, GalR1: galanin receptor-1, Gal/GalR1: Galanin to galanin receptor-1, HOMA2IR: Homeostatic model assessment (HOMA) of insulin resistance, HOMA%B: HOMA of beta-cell function, and HOMA%S: HOMA of insulin sensitivity*.

Table 6 shows the necessary data to train ANN for predicting the occurrence of LC. The control group included individuals who were in a state of optimal health. The ANN model included a 10-unit input layer and a normalization rescaling technique. There were two concealed layers: the first had two units and the second had three units. The hyperbolic tangent was employed as the activation function. The output layer employed two units with an identity activation function to distinguish between individuals with LC and those who are healthy. On the training set, the model showed remarkable prediction ability, with a sensitivity of 85.2 % and specificity of 94.4 %. The ANN model showed a 91.7 % sensitivity and a 100.0 % specificity for the prediction of LC in suspected subjects. Fig. 2 illustrates the relative significance of the top five independent variables, expressed as percentages: CRP (100.0 %), In/Glu (74.4 %), Gal (69.4 %), glucose (58.4 %), and GalR1 (33.3 %). The relevance of CRP was found to be the highest (100.0 %), demonstrating a robust correlation between CRP levels and the severity of LC.

**Table 6:** The Results of neural networks for prediction of Long-COVID using healthy controls as a reference group.

	Models	Long-COVID *vs.* Control
Input Layer	Number of units	10
	Rescaling method	Normalized
Hidden layers	Number of hidden layers	2
	Number of units in hidden layer 1	3
	Number of units in hidden layer 2	2
	Activation Function	Hyperbolic tangent
Output layer	Dependent variables	Patients *vs.* Control
	Number of units	2
	Activation function	Identity
	Error function	Sum of squares
Training	Sum of squares error term	5.099
	% Incorrect or relative error	11.1 %
	Prediction (sensitivity, specificity)	85.2 %, 94.4 %
Testing	Sum of Squares error	0.762
	% Incorrect or relative error	6.3 %
	Prediction (sensitivity, specificity)	91.7 %, 100.0 %
	AUC ROC	0.939
Holdout	% Incorrect or relative error	24.1 %
	Prediction (sensitivity, specificity)	71.4 %, 87.5 %

AUC ROC: area under the curve of receiver operating curve.

## 4 Discussion

Compared to the control group, patients with LC had a longer illness duration, a higher maximum body temperature, a longer recovery period, and a different treatment medication as found previously [41]. These findings suggest possible differences that may occur during the acute phase of SARS-CoV-2 infection, which could have a role in the development of LC symptoms. Disease severity, history of reinfection, underlying illnesses, and gender have been linked to LC in prior research [42]. Furthermore, azithromycin was independently linked to SARS-CoV-2 infection rates [43]. The acute phase of SARS-CoV-2 infection was crucial to the development of LC symptoms, but sociodemographic factors were less important.

Serum levels of Gal, GalR1, and Gal/GalR1 ratio were significantly increased in LC patients from the healthy control group (Table 2). This shows that there are changes in the Gal signaling system, which is known to have a role in both physiological and pathological processes such as inflammation and endocrine regulation [[44], [45], [46], [47]]. These alterations may contribute to the underlying mechanisms of LC. Increased level of Gal was associated with the presence of respiratory diseases such as obstructive sleep apnea syndrome [48]. The increase in Gal and its receptor (GalR1) can be illustrated through their antinociceptive effects [49], and endocrine regulation [50]. Hence, the increase may be a reaction from the body to reduce the pain. Also, GalR1 is widely distributed in different types of peripheral neurons and the brain [51,52]. These GalR1 affect cognitive control functions in rats through its direct actions in the ventral prefrontal cortex and the ventral hippocampus [53]. The findings demonstrate the unique neuromodulatory and behavioural roles of galanin, mediated by specific subtypes of neurones within the hippocampus and prefrontal circuits. LC is usually associated with the IR state [54,55], which is in turn related to the increase in Gal and GalR1 in animals [56,57], and human subjects [58,59]. A significant component that lowers IR and enhances glucose absorption in the diabetic heart is Gal [46]. It enhances food consumption and maintains control over blood pressure and energy balance in animals [60,61]. GalR1 exhibits a notable distribution in several regions of rats, including the hippocampus, thalamus, brainstem, stomach, heart, lung, kidney, muscle, and adipose tissue [56,57]. Gal has the potential to increase the responsiveness of insulin in rats through its three G protein-coupled subtype receptors [56,57]. There is growing evidence indicating that Gal suppresses insulin release from pancreatic islets. In vivo, administration of Gal inhibited basal insulin secretion in a dose-dependent manner [62]. Increasing evidence suggests that Gal inhibits the secretion of insulin from pancreatic islets. When given in a living organism, the introduction of Gal suppressed the release of insulin at its normal levels in a way that depended on the dosage [63,64]. Elevated plasma Gal levels were found in patients with obesity and diabetes [47,65]. In comparison to healthy controls, obese people had a considerably greater gal concentration. Furthermore, a direct association was observed between Gal and triglyceride (TG) levels in individuals with obesity. Higher levels of Gal may be used as a biomarker to help identify the onset of obesity [66]. There is a strong and positive association between the levels of serum Gal and fasting blood glucose, glycosylated hemoglobin, HOMA2IR, and TGs [67]. Gal exerts its actions by stimulating Gal receptors 1, 2, and 3. It has a crucial function in controlling energy equilibrium and adjusting food consumption [45,47,58,68]. It was observed that individuals with type 2 diabetes mellitus (T2DM), gestational diabetes mellitus, and obesity had considerably higher levels of Gal concentration [58,59].

In addition, LC patients exhibited elevated levels of glucose, insulin, HOMA2IR, HOMA%B, and HOMA%S, suggesting the presence of IR and disruptions in glucose metabolism in LC. These findings are consistent with other research that emphasizes the connection between COVID-19 and metabolic abnormalities, such as IR, elevated blood sugar levels (hyperglycemia), and disrupted carbohydrate metabolism [46,54]. LC patients have significantly higher levels of glucose, insulin, HOMA2IR, HOMA%B, and HOMA%S, which suggests that they have impaired glucose homeostasis and IR. Obesity, metabolic syndrome, and type 2 diabetes are known to cause IR, which may be linked to severe acute COVID-19 and LC [[69], [70], [71]]. IR and dysregulated glucose metabolism in LC patients may cause persistent symptoms and consequences.IR and COVID-19 may be correlated in both directions. Pro-inflammatory state, angiotensin-converting enzyme (ACE2) expression, and insulin signaling cascade disruption may cause IR in SARS-CoV-2 infection [[72], [73], [74]]. In contrast, IR and metabolic dysfunction may increase the likelihood of severe acute COVID-19 and LC [[74], [75], [76]]. These data show how metabolic variables affect COVID-19 etiology. Besides metabolic abnormalities, IR and dysregulated glucose metabolism can cause cardiovascular illnesses, neurocognitive deficits, and neuroinflammation [[77], [78], [79]]. These results imply that LC has systemic effects on several organ systems in addition to respiratory symptoms.

Gal was negatively correlated with SpO2, showing that lower oxygen saturation levels are associated with increased Gal, as presented in Table 3. This suggests that Gal dysregulation in LC patients is linked to the oxygenation state. Gal levels also correlated positively with age, showing that older LC patients have greater Gal levels. This shows age may cause Gal signaling dysregulation in LC. Gal correlated positively with I/G, insulin, and HOMA2IR, but negatively with HOMA%S. Elevated Gal levels in individuals with LC are associated with IR and impaired glucose metabolism. Several studies link Gal to glucose metabolic balance, insulin secretion, and IR [24,26,80]. IR characteristics also correlated with GalR1, albeit less significantly. GalR1 showed positive associations with insulin and HOMA2IR, and negative associations with HOMA%S. While the associations were not as strong, they do raise the possibility that GalR1 plays a role in IR. Further research is needed to confirm these links' importance. The literature confirms our findings that COVID-19 causes IR, hyperglycemia, and dysregulated glucose metabolism [80,81]. LC patients may have persistent symptoms due to metabolic problems connected to COVID-19 severity. Furthermore, Gal's significance in insulin secretion, glucose homeostasis, and IR has been emphasized in earlier research [26,82]. Given the link between Gal and SpO2, oxygenation status may contribute to the dysregulation of the Gal signaling pathway in LC. The causes and clinical implications of these discoveries need more study. Our study found strong relationships between Gal, GalR1, Gal/GalR1, sociodemographic, and metabolic parameters in LC patients. This suggests Gal signaling pathway changes, glucose metabolism, and IR disorders. These findings highlight the importance of metabolic variables in LC therapy and follow-up. The pathways relating to Gal, GalR1, and metabolic dysfunction in LC should be investigated, as a potential treatment.

The results of the multivariate GLM analysis suggest that LC is the main cause of elevated biomarker levels in accordance with previous results [17]. The stimulation of GalR2 by Gal and its agonists has demonstrated promise in reducing IR and depression-like behavior [83]. Moreover, the measurement of Gal levels has been suggested as a potential biomarker for predicting impaired glucose tolerance in clinical settings [27]. Our work shows that LC diagnosis significantly affects biomarkers, suggesting metabolic disturbances. These results show that LC should be seen as a separate illness with specific metabolic problems. Gal, IR, and associated biomarkers may help identify LC pathogenesis. They might monitor illness progression and guide treatment. These findings need to be confirmed and expanded by larger cohorts and longitudinal studies.

To utilize ANNs to forecast LC occurrence, with healthy controls serving as a comparative group (Table 6). ANNs demonstrated promising prediction accuracy and sensitivity, suggesting they may effectively diagnose the illness. It performed well on an independent dataset with 91.7 % sensitivity and 100.0 % specificity. In the holdout evaluation, the relative error rate was 24.1 %, suggesting the model's predicted performance may fluctuate with unseen data. These findings confirm prior neural network prediction studies regarding diabetes and obesity as risk factors for severe acute and LC due to IR [29]. Even after recovery, COVID-19 alters glycometabolic regulation, IR, and beta cell dysfunction, warranting additional study of metabolic anomalies in LC [30]. It needs larger and more diversified datasets to validate and refine its generalizability. The findings highlight the potential of ANN as an important diagnostic tool for LC and add to the expanding corpus of research on the subject. Collectively, these findings emphasize the capacity of these biomarkers to forecast and track the intensity of LC. Nevertheless, additional investigation utilizing more extensive and varied datasets is critical to authenticate and enhance these discoveries.

## 5 Limitations

A larger population sample is required to validate and generalzed our results. However, restricted subjects, uncontrolled variables, and a limited biomarker range limit the study's insights into LC's impact on biomarker levels and clinical features. Although some biomarkers show a good sensitivity for the differentiation between LC and control, these biomarkers are not too specific for LC and increased in other infection or inflammatory conditions. These are the major limitations of the present study.

## 6 Conclusions

LC patients had higher Gal, GalR1, and Gal/GalR1 concentrations than the control group, indicating Gal system changes. Also, IR was likely present in LC patients due to their increased glucose and insulin levels. The correlation analysis revealed a significant negative relationship between the levels of Gal, GalR1, and SpO2, but there was a positive association with age. Gal levels had a positive link with insulin, I/G, and HOMA2IR, while displaying a negative correlation with HOMA%S. The levels of GalR1 exhibited similar correlations with insulin and HOMA2IR and exhibited inverse correlations with HOMA%S. The findings indicate that LC significantly affects the concentrations of biomarkers, including those related to the Gal system and IR. Nevertheless, these findings augment our understanding of LC, aiding in developing diagnostic techniques and therapeutic strategies for affected individuals.

## CRediT authorship contribution statement

**Wasim Talib Al Masoodi:** Writing – review & editing, Writing – original draft, Visualization, Validation, Software, Resources, Project administration, Methodology, Investigation, Funding acquisition, Data curation, Conceptualization. **Sami Waheed Radhi:** Visualization, Validation, Resources, Project administration, Methodology, Investigation, Conceptualization. **Habiba Khdair Abdalsada:** Visualization, Validation, Resources, Project administration, Methodology, Investigation, Conceptualization. **Hussein Kadhem Al-Hakeim:** Writing – review & editing, Visualization, Validation, Resources, Project administration, Methodology, Investigation, Data curation, Conceptualization.

## Statement of ethics

The University of Kufa's institutional ethics committee evaluated and approved the research protocols before starting any investigations involving human participants (approval number: 1657/2023). All patients/participants gave their free and informed consent in writing before taking part in the study in compliance with ethical standards.

## Agreement to publication statement

All subjects gave written informed consent for the study's findings to be published before they participated in the research.

## Consent for publication

Before participating in this study, each subject provided written informed consent.

## A declaration on animal and human rights

The research was conducted by the ethical criteria outlined in the World Medical Association Declaration of Helsinki, as well as complying with Iraqi, global, and privacy regulations about research involving both humans and animals. The protocols and procedures that were implemented followed the guiding principles established by the Belmont Report, the CIOMS Guidelines, the International Conference on Harmonization in Good Clinical Practice (ICH-GCP), and the Declaration of Helsinki. In addition, our Institutional Review Board (IRB) protects participants' rights and welfare during the study by following the Guidelines for the International Protection of Human Research Subjects.

## Funding

There was no funding for this study. The authors of the submitted paper declare that they have no financial or other conflicts of interest with any organization.

## Declaration of competing interest

The authors declare that they have no known competing financial interests or personal relationships that could have appeared to influence the work reported in this paper.

## Data Availability

Data will be made available on request.
